# Turning the Tables—An Interview with Nicholas Wade

**DOI:** 10.1371/journal.pgen.0010045

**Published:** 2005-09-30

**Authors:** Jane Gitschier

For many of us, keeping up with the literature poses a never-ending challenge. We rely on word of mouth, “news and views,” journal clubs, or E-mail alerts to stay abreast of our field. But the media are also potent arbiters of scientific advances. And in the realm of genetics, I can think of no better source than Nicholas Wade of the *New York Times*.

**Figure:**
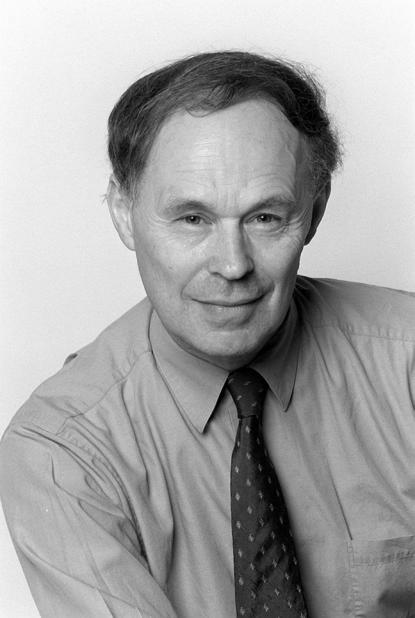
Nicholas Wade

What I like about reading Wade is that he gets right to the point—describing the discovery at hand in the first paragraph, yet roping me in for the full story. He's clear, crisp, and rarely misses the mark.

When visiting my father in rural Pennsylvania recently, I had the opportunity of interviewing Wade in situ at the venerable *Times* office building in Manhattan. The bus deposited me at the Port Authority Bus Terminal at the corner of 8th Avenue and 41st Street in blistering heat. I quickly walked two blocks north and turned the corner at 43rd Street to encounter the massive *Times* structure looming above, its signature globe lights forming a beacon to a set of revolving doors. I entered beneath the motto “All the news that's fit to print,” registered at the front desk, and took the elevator up to the fourth floor, where I was met by Wade.

Wade gave me a quick accounting of the enormous newsroom, which at that level, houses science and arts reporters and editors. On passing through a maze of cubicles, he commented that it looked just like any office building. “A lot messier,” I rejoined, as untidy stacks of papers were strewn everywhere. Inside a small conference room, I fiddled with my tape recorder, cognizant of a pro watching this neophyte. I looked across to a British man with a soft voice, lively blue eyes, and a puckish grin. He wasted no time turning the tables on me by asking, “Will the questions get too pointed?”

“No, they won't,” I replied. “I'm very sweet.”

“Your first mistake,” said he. And thus we began.

**Jane Gitschier:** How do you describe your beat?

**Nicholas Wade:** We're a small department, and the boundaries of the beats are flexible. By and large, I cover whatever I'm interested in. I manage to keep out of others' way because I keep close to the frontiers of biological research, particularly genetics and molecular biology, and no one else has quite the same interests. Many of my colleagues write about medicine, for example, but I write only about things that are perfectly useless, given that it takes some time to translate basic research into anything practical.

It's one of the challenges we have as a science section—to get people interested in things that are of purely intellectual consequence.

**Gitschier:** How do you envision your readership?

**Wade:** As a general policy, the newspaper is addressed to the intelligent and informed reader, but it's always with the idea of bringing news. We're not in the business of education. People can find general information from a dictionary or the Internet.

For the science section, although we should have the same readership as the main newspaper, I assume that readers have a certain amount of scientific knowledge or interest, and, of course, many readers of the section are scientists. So we can sometimes put in more technical detail than we would in the main newspaper.

**Gitschier: **The *Times* doesn't do a readership poll to ask how many people are actually reading Nicholas Wade's articles?

**Wade:** Our business office does do those polls, but they are kept secret from us. That's with the idea that the content of the newspaper should not be driven by polls or market results but rather by what the editors think is important.

**Gitschier: **How long have you worked for the *New York Times*?

**Wade:** Longer than I like to think! I came in 1981.

I was on the news section of *Science,* and before that with *Nature*. At both journals, I was mostly concerned with political stories that affected science. I found I had a great deal to learn when I came back to science writing.

**Gitschier: **And before that, were you a scientist yourself or a journalist in another area?

**Wade:** I guess I always wanted to be a writer, but I was interested in science, so I read science at university [Cambridge], though without any intention of becoming a scientist. I didn't plan to be a science writer either, but the two things came together. I got a job with *Nature* in London when I was quite young, and was sent off to the Washington office that *Nature* had just set up. After a few years, *Science* asked me to join them. I thought it would be fun to work with an American company. I worked for them for about ten years.

The *Times* asked me to join them as an editorial writer to cover science, technology, and medicine, and I did that for about ten years. Editorial writing is great fun, but it's rather a limited art form.

**Gitschier:** Can you describe editorial writing?

**Wade:** Editorials are unsigned pieces [on the editorial page] because they are intended to be the voice of the paper. That anonymity may give the writer's words extra authority, but the disadvantage is that you lose your byline, unfortunately. So as a writer, you essentially disappear from public view.

The only exception is when you advocate a position that the editors do not think should be the position of the paper. The piece is then called an editorial notebook and appears with your byline to make clear it's just your opinion, not theirs.

After writing editorials for ten years, I became science editor. That, too, is a job that deprives you of a byline because there is almost no time to write. The science editors handle both the science stories in the daily paper, and those in the weekly science section. As an editor, you get to see how the paper works, which is of great interest, but you cease reporting, and spend a lot of time improving other people's stories and attending meetings.

One of the great things about being a writer, particularly an editorial writer, is that you get to know a lot. The whole world comes through New York, and many people want to talk to the *Times*. Often the reporters and editors on the main paper are too busy to see them, so they end up talking to the editorial board, where the pressure of work is much less. So even if you don't cover foreign policy or defense, you can get to meet the leaders in these fields by sitting in on your colleagues' meetings.

But when I became an editor, I found I was one step back from the front line of the news. Being unable to research or write anything, my intellectual capital dwindled fast, until I began to feel I had gone from knowing almost everything about the world as an editorial writer to knowing almost nothing [as an editor]. On a newspaper, the most interesting job is reporting. I went back to writing as a reporter five or six years ago.

**Gitschier:** I'm interested in the process that you undergo in developing a story. First, how do you discover what's out there?

**Wade:** Mostly, we find out through the main journals that we watch. Most of them have now become sophisticated in preparing what they call “tip sheets,” or weekly lists of their most newsworthy articles, which are seen as a marketing tool for journals. They'll say to potential authors, “Send us your paper, and we'll get you mentioned in the press.” Tip sheets are useful but insidious because it's easy to rely on them too much and not read the stories in the rest of the journal. So it's an imperfect system.

**Gitschier:** What journals give you these tip sheets?

**Wade:** A lot of journals do it now. I look carefully at the *Nature* journals, *Science, PNAS [Proceedings of the National Academy of Sciences], American Journal of Human Genetics,* and the *Cell* journals.

**Gitschier:** And *PLoS,* of course!

**Wade:** *PLoS,* of course!

**Gitschier:** Are you ever strong-armed by anyone to write about his or her work?

**Wade:** People sometimes do call up, but not as much as I'd like. Scientists are reticent about promoting their work to the press because they risk being criticized by their colleagues for doing so. But sometimes people call to say, “I've got something very exciting,” and send me the paper in advance. It's always very useful to hear from people when they are enthusiastic about a result.

**Gitschier:** Do you attend scientific meetings?

**Wade:** Yes, I do, but not as many as I'd like. When you go to a meeting, you're usually obligated to write a story about it, and many scientific meetings are very hard to write about for the general reader because the findings are often incremental advances and difficult to summarize. On the other hand, meetings are very useful for talking to people in person, so I try to go to as many as I can.

**Gitschier:** After you read a paper, what's your next step?

**Wade:** I usually start by talking to the authors, and then call others in the field to see if they share the author's interpretation of the finding. Much of this can also be done with E-mail. I try to keep talking with people until I feel I understand a paper and its strengths and weaknesses, and then I'm ready to write it up.

**Gitschier:** Do you do most of your writing at home?

**Wade:** It's more restful to write at home. But if I have a story that will appear in the paper the next day, it's usually easier to be in the office.

**Gitschier:** How many articles do you write per week?

**Wade:** Usually about one or two, or more if there's lots of news. I've been on book leave for much of this year.

**Gitschier:** What is your new book about?

**Wade:** It's on what genetics is telling us about human evolution, human nature, and prehistory. I'm trying to integrate information from the many different fields that bear on the human past—paleoanthropology, archeology, historical linguistics, and evolutionary psychology—all of which are now being informed and amplified by genetics.

**Gitschier:** Back to the process. What's next?

**Wade:** You have to sell a story to your editor. It's a quite small department, so if a reporter says, “This is an important subject,” it will probably go into the paper. But the question is at what length because space is at a premium. The editors who run the main paper, who tend to have a political/foreign affairs background, may not be as enthusiastic about science as we are. The science department's editors have to assess how much space they are likely to get for a story.

**Gitschier:** I want to learn more about which stories you choose to develop. When you look back on the stories that you've written about over the past five to six years, which ones leap out at you?

**Wade:** The first that comes to mind is the race to sequence the human genome. It was a good science story, but it was also of interest to general readers because of the rivalry between Celera and the university people.

Another story I found interesting was the genetics of human dispersal. We've had the picture of human origins as developed by the paleoanthropologists, and it's wonderful how well they've done with the material they've had available to them—just a handful of skulls. Then, the geneticists arrived on the scene and added a whole new dimension.

For example, there was a paper by Mark Stoneking about when man first started to wear clothes. He managed to figure out the date at which the human body louse, which lives in clothing, evolved from the human head louse, which lives just in hair. And the date of that divergence must give the time at which humans first started to wear reasonably close-fitting clothing. It's wonderful that genetics can provide that quite surprising insight.

**Gitschier:** My favorite recent story is *Homo floresiensis*.

**Wade:** Isn't that nice! The referees took a whole year, I think, to convince themselves that this was real. They started off by thinking this fossil must be a pathological *Homo sapiens* skull, but then realized it doesn't look like *sapiens,* so it must be *erectus*. But it was found with artifacts just like those made by modern humans. To assume the little Floresians made these artifacts contradicts almost everything that paleoanthropologists have been taught: that we didn't start to make tools until our brains were about twice the size of chimpanzees', which are approximately the size of *Homo floresiensis*. This is such a paradoxical finding!

I think the paleoanthropology community is going through the same learning process as the reviewers did. They started with the assumption that these were modern human artifacts and a pathological skull, but eventually came to accept that everything was the work of a downsized *erectus*.

**Gitschier:** I like it when people are forced to rethink their dogma! What about the other side of the coin—stories that you missed?

**Wade:** I think the main one in that category is RNAi [RNA interference], about which I've written only one story. I kept thinking, “This is fascinating, but the general reader won't be interested in the details of molecular biology, so let's wait till it advances more.” I think I was far too late.

Another thing that is very difficult for science reporters to tackle is the fact that most scientific research ends nowhere. People can be very enthusiastic about what they are doing, but just as most drugs fail in clinical trials, many advances that seem very promising don't lead anywhere. So after you have been mistaken a certain number of times, you tend to be a little cautious. Of course, it's then very easy to become far more skeptical than one should be.

**Gitschier:** Gene therapy, for example, is a field that many thought had promise. It had some successes and some spectacular failures.

**Wade:** That's a field that's been going on for about 15 years. And almost all the coverage throughout the first ten years kept saying gene therapy is great. But in retrospect, it was quite wrong—it wasn't great at all. There were technical obstacles that have still not been overcome. I think the lesson for reporters is that they should not get too caught up in scientists' enthusiasm. It's fine to report that scientists are enthused about some new finding or project, but reporters should remain detached about whether or not it will succeed.

Stem cells are a case in point. The hidden premise of proposals for stem cell therapy is that we needn't understand exactly what is going on because if you just put the cells in the right place they will know what to do. My fear is that we need to understand the total cell circuitry to get stem cells to do anything useful, and that won't happen for years.

**Gitschier:** By choosing to write up certain stories and ignoring others, you are making judgments. Are there things out there that you are not writing about because you simply don't agree with them?

**Wade:** The only criteria that reporters are trained to apply is “Is this news?” So it doesn't matter if you agree with it or not.

**Gitschier:** But is that the ethical thing to do?

**Wade:** If someone makes a newsworthy claim that I suspect is not true, I will try to see if there are skeptics and expose readers to both sides of the issue. A reporter's job is to give readers sufficient information to make up their own minds. In a news story, you should expose people to all the possibilities, but you don't have to decide which one is correct. The hard thing about writing editorials is that you have to decide.

**Gitschier:** It's such a responsibility, I would think.

**Wade:** If you try to figure out the consequences of every article, you'd never write anything.

**Gitschier:** Returning then to the question of editorial writing, what were some of the memorable topics that you had to write about?

**Wade:** There weren't that many scientific issues about which we could have an editorial opinion, since many issues in science are a matter of ascertainable fact, not opinion.

I was writing editorials during the Reagan administration, so there were many environmental issues to inveigh about, inspired by the likes of Ann Gorsuch and James Watt. During the Reagan military buildup, I also wrote many editorials about military hardware and procurement scandals. I remember having great difficulty making up my mind about a “big science” project dear to physicists, the superconducting supercollider. I wrote one editorial in favor of it, the next year one against it, and the third year one in the middle. Editorial writers have to do their learning in public, or at least I did. Life is much easier once you have developed your position on the issues.

**Gitschier:** Today, there would seem to be a lot of opportunities for editorial writing in genetics—embryonic stem cells, cloning, reproductive choices, and intelligent design, to name a few.

**Wade:** I think you're right. Of course, stem cells would be less of an issue if the government hadn't tried to restrict the research. Intelligent design is a good subject for editorials, though not, I think, for the science section because it has no scientific content. It's a debate that was settled in the 19th century. It's not our role to educate people, and I see no more reason to discuss whether intelligent design is an alternative to evolution than to discuss whether or not the earth is flat.

**Gitschier:** What about the urgency to write things?

**Wade:** There is nothing like a deadline for concentrating your mind. Some of the hardest stories are when you are asked to get a story at very short notice, such as late at night when the editors see the *Washington Post* has some story, and ask you to match it. If you don't have the home numbers of the people you need to talk to, you're out of luck. Most reporters know their beat well enough that they can match a story at short notice. Fortunately, it doesn't happen too often.

**Gitschier:** One of the things I like about my job as a geneticist is that there is always something new on the horizon. You must feel the same way.

**Wade:** Yes, and journalists have the luxury of being able to move from one field to another. If it's a slow week in genetics, I can write about cognitive science.

If you're not learning something new every day, you have no one but yourself to blame.

